# Feasibility of hepatocellular carcinoma treatment based on the tumor microenvironment

**DOI:** 10.3389/fonc.2022.896662

**Published:** 2022-09-13

**Authors:** Haiqiang Wang, Fan Shi, Shudan Zheng, Mei Zhao, Zimeng Pan, Li Xiong, Lihong Zheng

**Affiliations:** ^1^ Department of Internal Medicine, First Affiliated Hospital, Heilongjiang University of Chinese Medicine, Harbin, China; ^2^ Graduate School of Heilongjiang University of Chinese Medicine, Harbin, China; ^3^ Department of Internal Medicine, Fourth Affiliated Hospital, Heilongjiang University of Chinese Medicine, Harbin, China

**Keywords:** hepatocellular carcinoma, tumor microenvironment, immunotherapy, intestinal microorganisms, oncolytic viruses, anti-vascular proliferation

## Abstract

The incidence of liver cancer is extremely high worldwide and poses a serious threat to human life and health. But at present, apart from radiotherapy, chemotherapy, liver transplantation, and early resection, sorafenib was the main systemic therapy proven to have clinical efficacy for unresectable liver cancer (HCC) until 2017. Despite the emerging immunotherapy in the past decade with immune inhibitors such as PD - 1 being approved and applied to clinical treatment, there are still some patients with no response. This review aims to elucidate the mechanisms underlying the tumor microenvironment of hepatocellular carcinoma and thus analyze the effectiveness of targeting the tumor microenvironment to improve the therapeutic efficacy of hepatocellular carcinoma, including the effectiveness and feasibility of immunotherapy, tumor oncolytic viruses and anti-vascular proliferation therapy.

## Introduction

Liver cancer is one of the most common and deadly malignancies worldwide (1), and hepatocellular carcinoma accounts for 90% of all liver cancers (2), and is an abnormal and malignant proliferation of liver cells, with an estimated one million cases of liver cancer per year by 2025 (3). Hepatocellular carcinoma often develops in the context of underlying liver injury (4), and is closely associated with chronic liver disease. Patients with chronic liver disease are often accompanied by liver inflammation, fibrosis and abnormal hepatocyte regeneration, and these abnormalities may lead to cirrhosis, and cirrhosis increases the risk of hepatocellular carcinoma (1). Risk factors for liver cancer are extensive and include HBV infection, HCV infection, aflatoxin B1 exposure, excessive alcohol intake, non-alcoholic fatty liver, diabetes mellitus, obesity, smoking etc. (5). Surgery is the most effective treatment (6), ultrasound combined with serum AFP test is sensitive and specific for early stage liver cancer surveillance and specificity is high (7). If detected at an early stage, it can be treated invasively (8),however, most patients are diagnosed only when the tumor is too advanced to be treated by surgical resection, *in situ* liver transplantation or local percutaneous tumor ablation (9), thus leading to a poor prognosis for hepatocellular carcinoma. Local therapy is the most common first-line treatment methods, including percutaneous local ablation, chemoembolization, radioembolization, and external irradiation therapy. Arterial embolization can be used for patients with tumors that are not amenable to radical resection or ablation, without extrahepatic spread and with intact liver function (9). For patients with unresectable hepatocellular carcinoma, The tyrosine kinase inhibitor (TKI) sorafenib is the primary approved systemic therapy as of 2017 (10). Although the clinical treatment of HCC has improved greatly in recent years, the prognosis is relatively poor, due to the lack of efficient treatment for hepatic malignancies and due to the complexity of the tumor microenvironment. For patients with advanced diagnosis of HCC, the survival rate is not high, so further research and analysis are still needed to find a better treatment for hepatocellular carcinoma.

The tumor microenvironment is the site of rapid tumor progression. Various factors in the tumor microenvironment cause abnormal vascular proliferation and immunosuppression, leading to rapid progression of hepatocellular carcinoma. By targeting the tumor microenvironment, and applying immunotherapy alone or in combination with immunoregulation, the state of immunosuppression is transformed into the state of immune stimulation to kill tumor cells. Lysozyme virus directly destroys tumor cells, but also modulates immunity and destroys the tumor vascular system. Anti-vascular endothelial growth factor inhibitors are applied to inhibit abnormal vascular proliferation and block tumor cell nutrient supply, alleviating immunotherapy resistance. These therapies have shown satisfactory efficacy in the treatment of hepatocellular carcinoma and have expanded the idea of hepatocellular carcinoma treatment. This essay searched the PubMed database for the mechanisms of tumor microenvironment generation and the treatment of hepatocellular carcinoma in the past decade, and summarizes the mechanisms and clinical applications of emerging immunotherapies, oncolytic virus therapies and anti-vascular proliferation therapies in recent years.

## Tumor microenvironment

The tumor microenvironment is the cellular environment of tumorigenesis, which is involved in regulating the occurrence, development, invasion and metastasis of malignant tumors, and plays a very important role in the development of hepatocellular carcinoma (HCC).

Hypoxia in the tumor microenvironment is thought to be an important driver of hepatocellular carcinoma progression (11). Hypoxia arises from insufficient blood supply due to the combination of excessive proliferation of malignant cells and insufficient vascularization during tumor cell progression (12). Hypoxia can further promote malignant cell proliferation, and experimental results have demonstrated that tumor cells activate PI3K/AKT signaling pathway under hypoxia (13), leading to malignant over proliferation and radiotherapy resistance of cancer cells. Hypoxia also affects immune cells, reconstitutes the tumor immune microenvironment (TIM), suppresses the expression of immune T cells and NK cells, and promotes the expression of immunosuppressive cytokines (12). For example, activation of hypoxia-inducible factor 1*α* can upregulate PD-L1 expression (14), creating an immunosuppressive environment, thus protecting tumor cells from recognition and clearance by the host immune system, and ultimately leading to tumor escape and immune tolerance.

Abnormal proliferation of blood vessels in the tumor microenvironment is another major risk factor for the progression of hepatocellular carcinoma. HCC is a highly angiogenic cancer (15), angiogenesis plays a large role in tumor growth, early metastasis, and poor survival. The tumor microenvironment (TME) system is complex and consists mainly of cellular and non-cellular components. Cellular components including hepatic stellate cells, fibroblasts, immune cells and endothelial cells (ECs). Non-cellular components include growth factors (such as fibroblast growth factor (FGF), hepatocyte growth factor (HGF) and vascular endothelial growth factor (VEGF)), protein hydrolases, extracellular matrix (ECM) proteins, and inflammatory factors (16). Activated hepatic stellate cells secrete angiogenic growth factor, which together with vascular endothelial growth factor (VEGF) stimulates angiogenesis, forming a new vascular system within the TME (17) and providing various nutrients for tumor growth.

In addition, hepatic stellate cells are activated in the presence of liver injury and secrete large amounts of transforming growth factor-*β* (TGF-*β*), a key immunosuppressive cytokine involved in liver regeneration, inflammation and fibrosis, promoting fibrosis, cirrhosis, and ultimately liver cancer (18). Activated hepatic stellate cells recruit Tregs by suppressing lymphocytes, overexpressing PD-1 cells and promoting immune tolerance, and inhibits the activation of CD8+ T cells by reducing the IL -2/IL-2R T cell signaling pathway and promoting the production of myeloid-derived suppressor cells (MDSC) through the mediation of CD54 (18). Tregs cells as well as myeloid-derived suppressor cells (MDSC) are considered to be immune cells that promote tumor growth in the tumor microenvironment (19), and thus these are critical for tumor progression, metastasis and invasion.

Another player in TME is exosomes, small vesicular structures that act as communication mediators between cancer and non-cancer cells in the tumor microenvironment (20), containing multiple components such as DNA, RNA and proteins (15). These substances are involved in the growth and metastasis of hepatocellular carcinoma, promote angiogenesis, regulate the inflammatory microenvironment, evade immune surveillance (16), and promote tumor development. For example, Exosome MIRs induce epithelial-mesenchymal transition as well as angiogenesis, which are involved in different processes of hepatocellular carcinoma metastasis (21). And it has been demonstrated that miR-32-5p, delivered by drug-resistant cellular exosomes activates the PI3K/Akt pathway, which leads to multidrug resistance in hepatocellular carcinoma through angiogenesis and EMT, and becomes another obstacle to hepatocellular carcinoma treatment (22). Additional features of TME are low pH and the accumulation of adenosine, which favors tumor cell progression while being inhibitory to immune cells (12), thus participating in the development of an immunosuppressed state. It is worth to mention that exosomes are also considered as therapeutic vectors, and the delivery of miR-150-3p-rich exosomes to HCC cells may have therapeutic applications (23).

**Figure 1:**
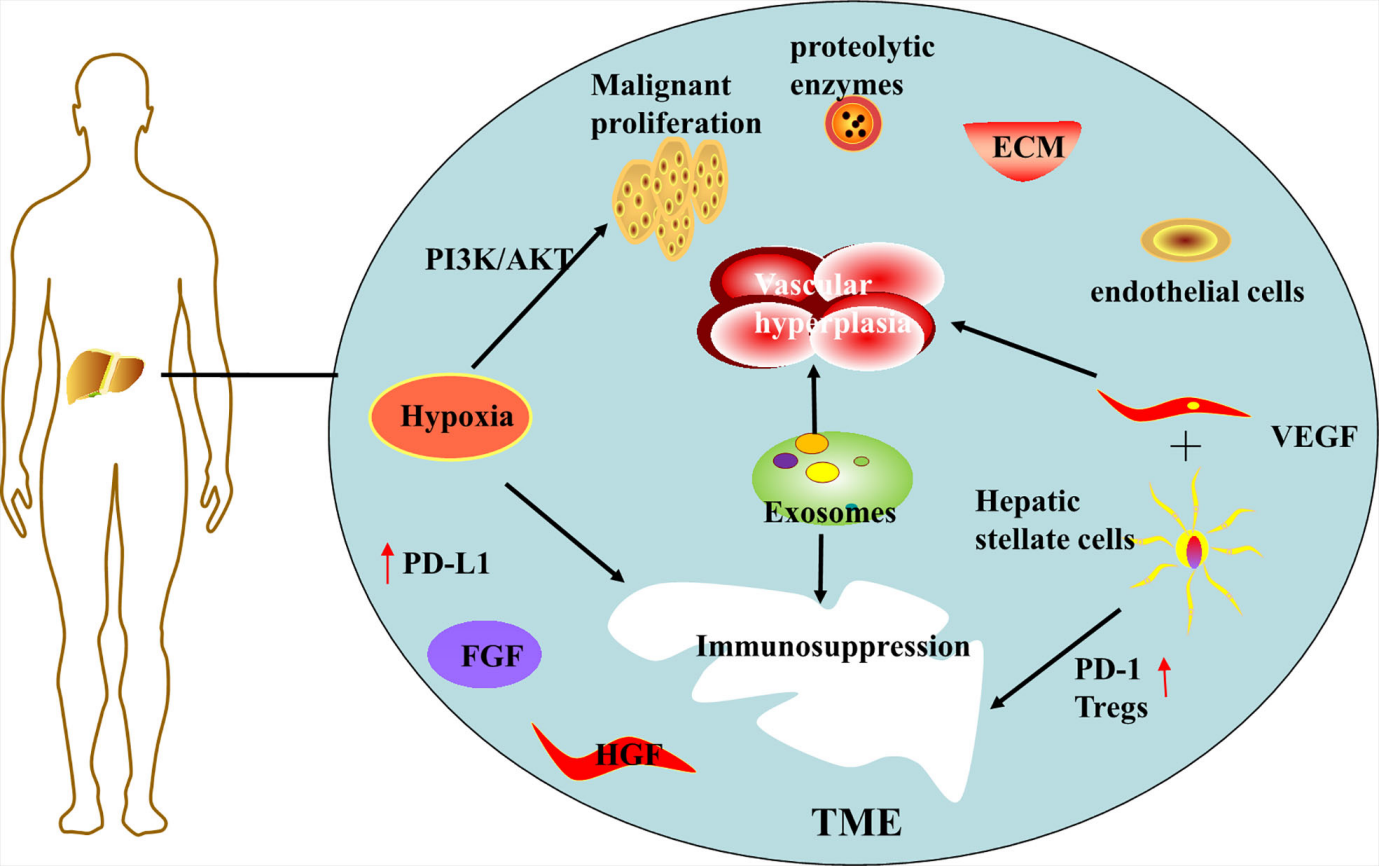
Schematic diagram of tumor microenvironment formation mechanism.

To briefly summarize, various factors in the tumor microenvironment cause abnormal vascular proliferation and immunosuppression, resulting in hepatocellular cell carcinoma progressing rapidly in the tumor microenvironment (**Figure 1**). Therefore, in the treatment of hepatocellular carcinoma, targeted interventions can be made to address the characteristics of the tumor microenvironment.

## Immunomodulatory therapy

Because the tumor microenvironment is in a state of immunosuppression and protects tumor cells from escaping and from the attack of immune cells, to control and treat liver cancer, immunity should be regulated and the immunosuppressive environment should be reversed. Immunotherapy is gaining worldwide acceptance as a new standard of care for hepatocellular carcinoma (HCC). Using targeted cytotoxic T Immune checkpoint inhibition of lymphocyte-associated protein-4 (CTLA-4) and anti-programmed cell death protein-1 (PD-1) cancer immunotherapy with pharmaceutical preparations (ICIs) (24), changing the traditional sorafenib treatment mechanism, and as an adjuvant therapy to a certain extent, the recurrence rate has been reduced (25), expanding the treatment ideas for liver cancer and improving the survival rate (26).

PD-1 is an important immunosuppressive checkpoint molecule, mainly expressed on the surface of activated T cells, B cells and NK cells. The binding of PD-1 and its ligand PD-L1 inhibits the activation of T cells (27), decreases autoimmunity and protects tumor escape. PD-1/PD-L1 immune checkpoint blockade enhances the immune function of tumor-specific CD8+ T cells for immune attack on tumors (28). Currently, PD-1 monoclonal antibody nivolumab, Pembrolizumab has been approved by the FDA as a second-line treatment for sorafenib failure (26). Nivolumab also prove the efficacy and safety in the treatment of unresectable HCC (29). In addition, several anti-pd-1 antibodies tislelizumab, camrelizumab and anti-PD-L1 monoclonal antibodies durvalumab, atezolizumab, avelumab have also shown more satisfactory efficacy in clinical trials (30).

CTLA-4 is a protein receptor expressed mainly on T regulatory (Treg) cells. Treg cells, a subset of CD4+ T cells, can block T cell responses, and blocking CTLA-4 reverses the suppression of T cell activation signaling, making it a potential immunotherapeutic approach (31). The anti-CTLA-4 monoclonal antibodies tremelimumab, ipilimumab is being continuously investigated in the treatment of HCC. A small phase II lead trial (NCT01008358) of the anti-CTLA-4 monoclonal antibody tremelimumab was tested in HCV-infected patients with advanced HCC and showed good partial response (PR) and stable disease (SD) rates and was well tolerated (32).

In addition to PD-1/PD-L1 and CTLA-4, it is essential to explore some new immune checkpoints. LAG3, TIGIT, TIM-3, VISTA, B7-h3, BTLA, have been shown to be promising therapeutic targets that may have opportunities for clinical application in the future (33). Particularly LAG3, as inhibition of LAG3 not only activates CD8+ cytotoxic T cells but also downregulates immunosuppressive regulatory Treg cells (31). PVRL1/TIGIT pathway plays an important role in HCC progression role, and TIGIT is a promising target against PD1 inhibitor resistance (34). TIM-3 is expressed in tumor cells and immune cells. The interaction of TIM-3 with its ligand has been shown to induce T cell suppression. Therefore, blocking TIM-3 expression leads to Tcell proliferation and cytokine production, which triggers immune activation (35). In addition, co-expression of TIM3 and PD1 makes it another attractive target for targeted cancer immunotherapy, and co-blockade of TIM3 and programmed cell death1 (PD1) can lead to a reduction in tumor volume in preclinical models, warranting further study in the clinic (36).

Targeted agents and checkpoint inhibitors are the only drugs approved for systemic treatment of advanced HCC (37). Despite the remarkable clinical success of immune checkpoint therapy, with significant clinical efficacy found for CTLA-4 and PD-1, low response rates and the development of drug resistance in some patients remain issues that need to be addressed. Hypothesized that one of the main reasons for ineffective and resistant PD-1/PD-L1-targeted immunotherapy is that the regulation of PD-L1 is influenced by multiple. For example, in recent studies, USP22 was found to strongly interact with PD-L1 *in vitro* and *in vivo*, inducing PD-L1 deubiquitination, thereby preventing proteasomal degradation of PD-L1 and stabilizing its protein expression levels, counteracting the effects of anti-PD-L1 drugs (38). USP22 is an identified oncoprotein that is highly expressed in hepatocellular carcinoma (HCC) but not in other types of cancer. USP22 can promote multidrug resistance (MDR) in hepatocellular carcinoma cells by activating the SIRT1/AKT/MRP1 pathway, which contributes to tumorigenesis and progression of hepatocellular carcinoma. This gives us a hint that USP22 may be a potential target that could reverse multidrug resistance (MDR) in HCC in the clinic (39). MEF2D promotes tumor growth, metastasis and angiogenesis, affects tumor cells and even the tumor microenvironment, increases PD-L1 expression in HCC cells, and suppresses CD8+ T cell-mediated antitumor immunity. SIRT7 blockade can reduce the dual effect of PD-L1 on hepatocellular carcinoma cell proliferation and decrease anti-tumor immunity through MEF2D regulation, providing a basis for the development of combined SIRT7 inhibitors and anti-pd -(L)1 drugs for the treatment of hepatocellular carcinoma (40). This is a direction worth investigating in the future. It also suggests that immune combination applications are likely to be an effective measure to improve this situation.

### Combination of PD-1/PD-L1 inhibitors and CTLA-4 inhibitors

Combination immunotherapy enhances the anti-tumor effects of PD-1/CTLA-4 dual blockers (41). Nivolumab + ipilimumab and durvalumab + tremelimumab are currently approved by the FDA for the treatment of patients with advanced HCC and have achieved better clinical outcomes compared to single agents (26). Nivolumab + ipilimumab is a widely studied combination immunotherapy (42). Data published in ASCO 2019 showed that the anti-Pd-1 antibody nivolumab combined with the anti-CTLA -4 antibody ipilimumab induced complete pathological remission within 6 weeks in 29% of patients with resectable HCC (43).

### Immunotherapy combined with MKIs

MKIs such as sorafenib, regorafenib and sunitinib are now used in first and second line treatment of HCC. Their mechanism of action targets multiple kinases by inhibiting various proteins of the VEGF receptor, platelet-derived growth factor, STAT3 and kinase cascades (43). Tyrosine kinase MET is considered an excellent target for hepatocellular carcinoma treatment (44). However, the efficacy of

sorafenib is limited by the development of drug resistance, the major neuronal isoform of RAF, BRAF and MEK pathways play a critical and central role in HCC escape from TKIs activity. A possible strategy could be the combination of RAS/RAF/MEK/ERK pathway inhibitors with other pathways inhibitors, But further clinical studies are needed (45). The growth of HCC cells after sorafenib resistance has been shown to be ameliorated using dual inhibition of Akt and Met, enhancing the effect of sorafenib, but has not been evaluated in patient-derived xenografts (46), and the HGF/MET axis is also considered to be an important pathway for tumor treatment (47). The combination of immunotherapy with tyrosine kinase inhibitors MKIs has been increasingly explored in recent years. Experiments by Li et al. found that MET-mediated phosphorylation and activation of GSK3B resulted in reduced PDL1 expression, and that the combination of anti-PD1 and anti-PD-L1 with MET inhibitors, such as the MET inhibitors tivantinib and capmatinib, increased PD-L1 expression. And compared with treatment with MET inhibitor or anti-pd1 alone, the duration of both drugs significantly inhibited hepatocellular carcinoma cell growth and prolonged survival time in mice. Treatment of HCC mice with sunitinib in combination with anti-PD-1 resulted in better treatment response and more pronounced tumor regression (43).

### Immunotherapy combined with regulation of intestinal microbes

The human intestinal microbiota consists of a complex community of microorganisms, the largest micro-ecosystem in the human body, including archaea, bacteria, viruses, fungi, etc., which work together to regulate nutrition, metabolism and immunity (48). The intestine and liver share a common origin in the foregut, and although the liver has no direct contact with intestinal microorganisms, it has a close relationship through the biliary tract, hepatic portal vein, and bile secretions that coordinate and interact with each other (49), and play a vital role in disease and health status. Growing evidence from experimental and clinical studies suggests that gut microbes play an important role in the development and treatment of liver cancer (50). First, during HCC development and progression, intestinal microorganisms promote the formation of the tumor microenvironment (TME), with the main mechanisms being dysbiosis and leaky gut (51). Dysregulation results in a more permeable intestinal barrier, and a leaky gut allows bacterial metabolites and microbial associated molecular patterns (MAMPs) to translocate and reach the liver (8). It was also found that in China, patients with persistently elevated total serum bile acids had a significantly higher risk of developing HCC, and that bile acids may play an important role in the progression of the underlying liver disease that leads to liver cancer (52). Bound primary bile acids are associated with an increased risk of HBV and HCV-associated HCC, but higher secondary bile acid levels are not associated with an increased risk of HCC (53), corroborating the link between bile acids and hepatocellular carcinoma.

Promisingly, the use of antibiotics, prebiotics and probiotics can be used to regulate intestinal flora and prevent the development of liver cancer (54). Fecal microbiota transplantation (FMT) has been shown in mice to restore intestinal flora diversity and reduce the risk of nonalcoholic steatohepatitis (NASH) developing hepatocellular carcinoma (HCC) (55). Despite the lack of data on the impact of FMT on HCC, fecal microbiota transplantation could be a potential treatment option for NAFLD/NASH progression and could be considered as an augmentation strategy with immune checkpoint inhibitors applied together. Host response to ICIs (PD-1/PD-L1 blockade or CTLA-4 inhibition) may be influenced by the composition of the gut microbiome (48). Stool specimens from immune-responsive patients had higher intestinal flora diversity than specimens from non-responsive patients diversity of intestinal flora (56). Intestinal flora can indirectly affect PD-1 and PD-L1 expression through local or systemic modulation of immune responses, enhancing the antitumor efficacy of PD-1 and PD-L1 blockade therapy (57). The gut microbiota may influence the antitumor immune response through innate and adaptive immunity, but the effect of the gut microbiota on the immune checkpoint inhibitor response has not been validated in HCC and needs to be extensively studied (58).

In addition, combination immunotherapy with CAR-T cells and checkpoint blockade is thought to be the next immunotherapy frontier as it provides the two elements necessary for strong immune responses: CAR-T cells, which provide the infiltrate and PD-1/PD-L1 blockade, which can ensure sustained T cell persistence and function (59). Immunotherapy can also be combined with other local treatments, such as combined local ablation, local radiation therapy, transcatheter arterial chemoembolization (TACE), etc. Local treatment not only destroys the primary tumor, but also stimulates the release of tumor antigens, thus improving the efficiency of immune response in liver cancer (60). A number of clinical trials of immunotherapy and topical treatment clinical trial studies are also underway (61). Although, the clinical efficacy of immunotherapy is very promising, clinical immune-related adverse events (IRAE), and the lack of prognostic markers are still non-negligible issues that need further clinical exploration in the future (62).

## Use of oncolytic viruses

Viral therapy was first applied in the 19th century, and was introduced as a treatment for cancer due to the observation that tumors appeared to regress after infection with viruses and the consideration that viruses might have a therapeutic effect on tumors (63). Oncolytic viruses can be divided into two broad categories, those that occur naturally and those that have been genetically modified by humans. Naturally occurring OVs include eutherovirus (Reo), Newcastle disease virus (NDV), enterovirus and measles virus (MV), and microvirus H-1 (H-1PV or Parvoryx), which are used in their native form.On the other hand, human modified viruses, such as herpes simplex virus (HSV), adenovirus (Ad), and cowpox virus (VV), are genetically modified viruses (64).

### Targeted regulation of tumor microenvironment by oncolytic viruses

**Figure 2:**
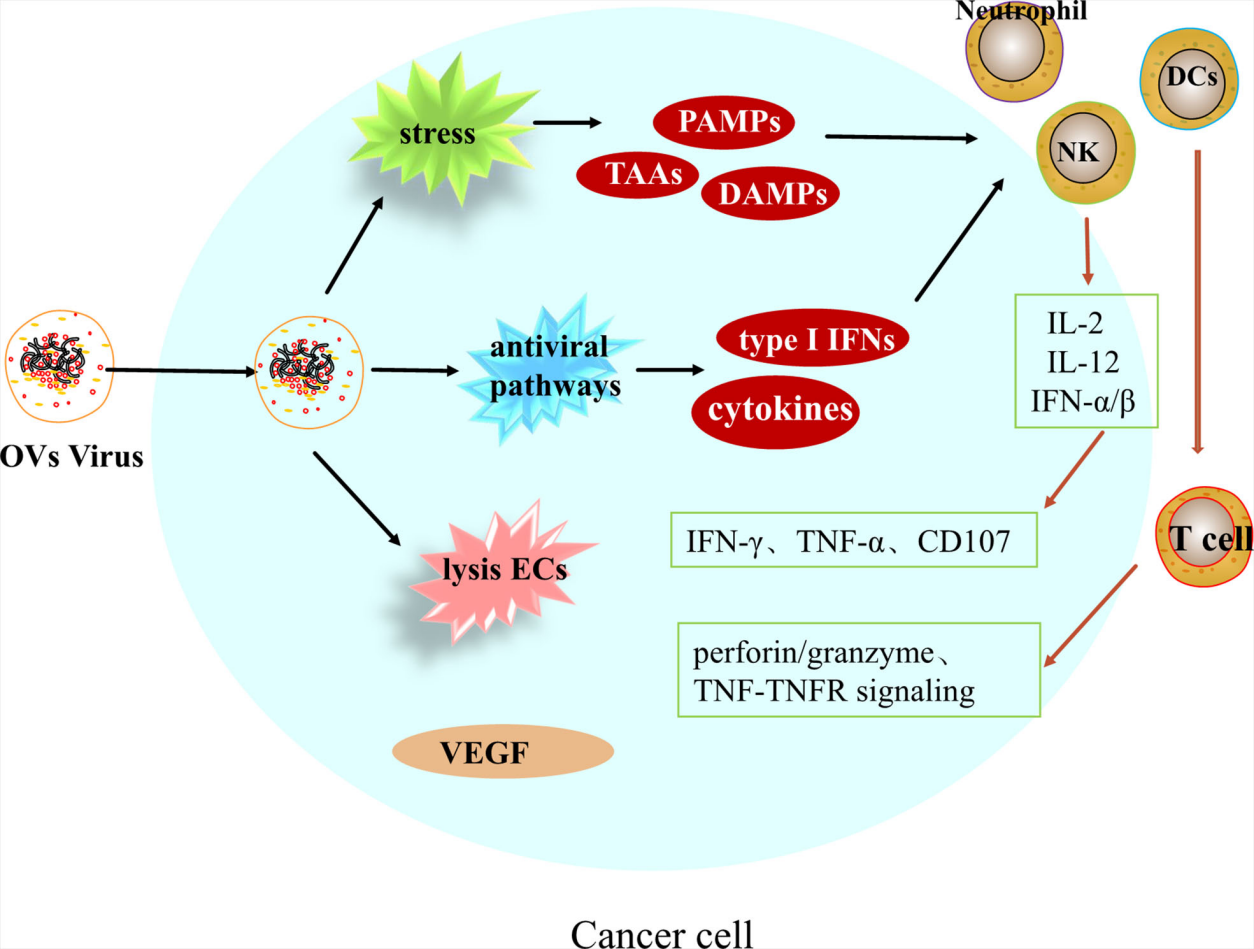
Multiple effects of oncolytic viruses on the tumor microenvironment.

Oncolytic viruses (OVs) are a class of biological agents with tumor-selective and replication capabilities (65). This therapy is a new and promising treatment for many different types of cancer. Oncolytic viruses is able to selectively replicate and destroy tumor cells, causing tumor cell lysis and subsequent release of viral progeny and tumor cell components, and is able to leave healthy cells unharmed (66). In addition to direct and specific destruction of tumor cells, Oncolytic viruses can also modulate immunity as well as disrupt the tumor vascular system, with multiple effects on the tumor microenvironment (**Figure 2**).

#### Induction of immune response

After entering tumor cells, OVs can induce systemic anti-tumor immune responses and induce innate and adaptive immune responses. Upon infection of hepatocellular carcinoma cells by OVs, viral replication leads to endoplasmic reticulum stress and genotoxic stress in cancer cells, releasing tumor-associated antigens TAAs, pathogen-associated molecular pattern molecules PAMPs and damage-associated molecular pattern molecules DAMPs, enhancing the activation of antigen presenting cells (APCs), which leads to the activation of immune cells such as dendritic cells, natural killer cells, macrophages and neutrophils, and inflammatory signaling (67). On the other hand, due to viral replication, activation of antiviral pathways, induction of cytokines and type I IFN, together mediating the activation of immune cells. Activated immune cells, NK cells, in the presence of chemokines such as IL-12, IL-2 and IFN-*α*/*β*, metastasize to the tumor area and release IFN-*γ*, TNF-*α* and CD107 to exert anti-tumor effects. Mature dendritic cells can initiate T cells in the background of MHC I and II molecules cells, triggering CTL killing of tumor cells through TNF-TNFR signaling, perforin/granzyme pathway. Regarding the regulation of adaptive immunity, according to Twumasi-Boateng et al. it is believed that oncolytic viruses are involved in the entire process of T cell initiation, transport, infiltration, activation and eventual killing of tumors, ultimately reversing immunosuppression and creating a micro-realm of immune stimulation. Therefore, the combination of OVs with tumor immunotherapy can overcome the immune inhibition in TME, thus greatly improving the effect of anti-cancer treatment (68, 69). But there is an important issue, and the number of potential combinations with immunotherapy is enormous, and which combination is most effective requires ongoing research (70).

#### Disruption of tumor vascular system

There is evidence that poxvirus strains are able to directly destroy infected tumor-associated endothelial cells and replicate within their system, leading to vascular collapse. In a phase II clinical trial, JX-594, a transgenic expression of a recombinant Wyeth poxvirus strain, was used in patients with hepatocellular carcinoma and showed that JX-594 caused acute tumor vascular rupture and reduced tumor perfusion in these patients and was maintained for at least 8 weeks, with no toxicity to normal blood vessels or wound healing noted (71).

In addition to promoting tumor vessel collapse, oncolytic vaccinia virus has recently been found to have antiangiogenic effects. By directly lysing tumor-associated endothelial cells (ECs), oncolytic viruses can reduce the level of vascular endothelial growth factor (VEGF) and thus exert anti-angiogenic effects. Vascular endothelial growth factor (VEGF) levels were significantly reduced in infected tumors after viral treatment, and VEGF production was also reduced in adjacent uninfected cells; therefore, a combination of oncolytic viruses and additional anti-angiogenic therapy may improve treatment outcomes (72).

### Clinical application of oncolytic viruses

Reo (73) is a member of the family Reooviridae and is an envelope-free double-stranded RNA virus (64). Induction of interferon (IFN) secretion and innate immune activation in human primary liver tissue in the absence of cytotoxicity and independent of viral genome replication. Meanwhile, Reo-induced cytokine response can effectively inhibit HCV replication and is supported by its clinical potential as a combined antiviral and antitumor therapy in HCC caused by HCV virus infection (74). It is worth noting that some studies have shown that to avoid potential side effects, try to avoid taking oral (75).

Cowpox virus (VV), a double-stranded DNA virus, is currently the most widely studied OVs for the treatment of hepatocellular carcinoma, and its mutant Pexa-Vec, also known as JX-594, is currently being evaluated in a phase III clinical trial in hepatocellular carcinoma (NCT02562755) (65). Preclinical studies of hepatocellular carcinoma lysing herpes simplex virus (oHSV) show that oHSV is highly selective for killing hepatocellular carcinoma (76).

However, to date, only three OVs have been approved globally for the treatment of advanced cancer (77). Despite the multi-mechanism therapeutic effect of OVs, the number of patients fully responding to OV monotherapy is small, so the effect of monotherapy is limited. It is continuously proven that the combination of OVs with other treatment modalities can unlock the therapeutic potential and improve the therapeutic efficacy (75). In addition to the combination of immunotherapy and anti-angiogenesis inhibitors we mentioned earlier, epigenetic dysregulation also plays a key role in hepatocarcinogenesis by altering gene expression through various mechanisms (78), so the combination of epigenetic modulators can also be considered (63). In addition to this, it can be used in combination with pericyte transfer (ACT), chimeric antigen receptor T cells (CAR-T) (79), bispecific T cell conjugates (BiTEs), and cancer vaccines (69).

### Efficacy and safety of oncolytic viruses

OVs are a drug with great therapeutic potential, but there are still many issues that need to be addressed, such as viral transmission, dosing, antiviral immunity, etc. (80). In solid tumors, OVs must bypass a series of barriers to reach the tumor site, so overcoming the physical barriers of the tumor microenvironment such as the extracellular matrix (ECM) to viral delivery is a great challenge. ECM consists of proteoglycans that can block the anticancer drug in solid tumors distribution. Therefore, during treatment, ECM degrading enzymes including collagenase and hyaluronidase can be administered to achieve ECM reorganization and promote the spread of the virus within the tumor on the one hand, and OVs expressing ECM degrading enzymes can be designed for use on the other hand. Pre-existing immunity to the virus also reduces the effectiveness of oncolytic viruses therapy and can be circumvented by increasing the dose of systemic administration of OVs and co-administration of cyclophosphamide (64). In order to better target hepatocellular carcinoma with oncolytic viruses, it has been demonstrated that the use of a cationic galactosylated polymer (Gal32-b-Agm29) as a vector allows systemic delivery of oncolytic viruses in hepatocellular carcinoma cell lines. OVs complexed with Gal32-b-Agm29 enables easier entry of viral cells into hepatocellular carcinoma cells, enhances viral replication, and ultimately leads to hepatocellular carcinoma cell lysis and the occurrence of a higher immunogenic cell death induction program (81). More future research is needed on how to safely address other clinical studies.

## Anti-anomalous proliferation of blood vessels

**Figure 3:**
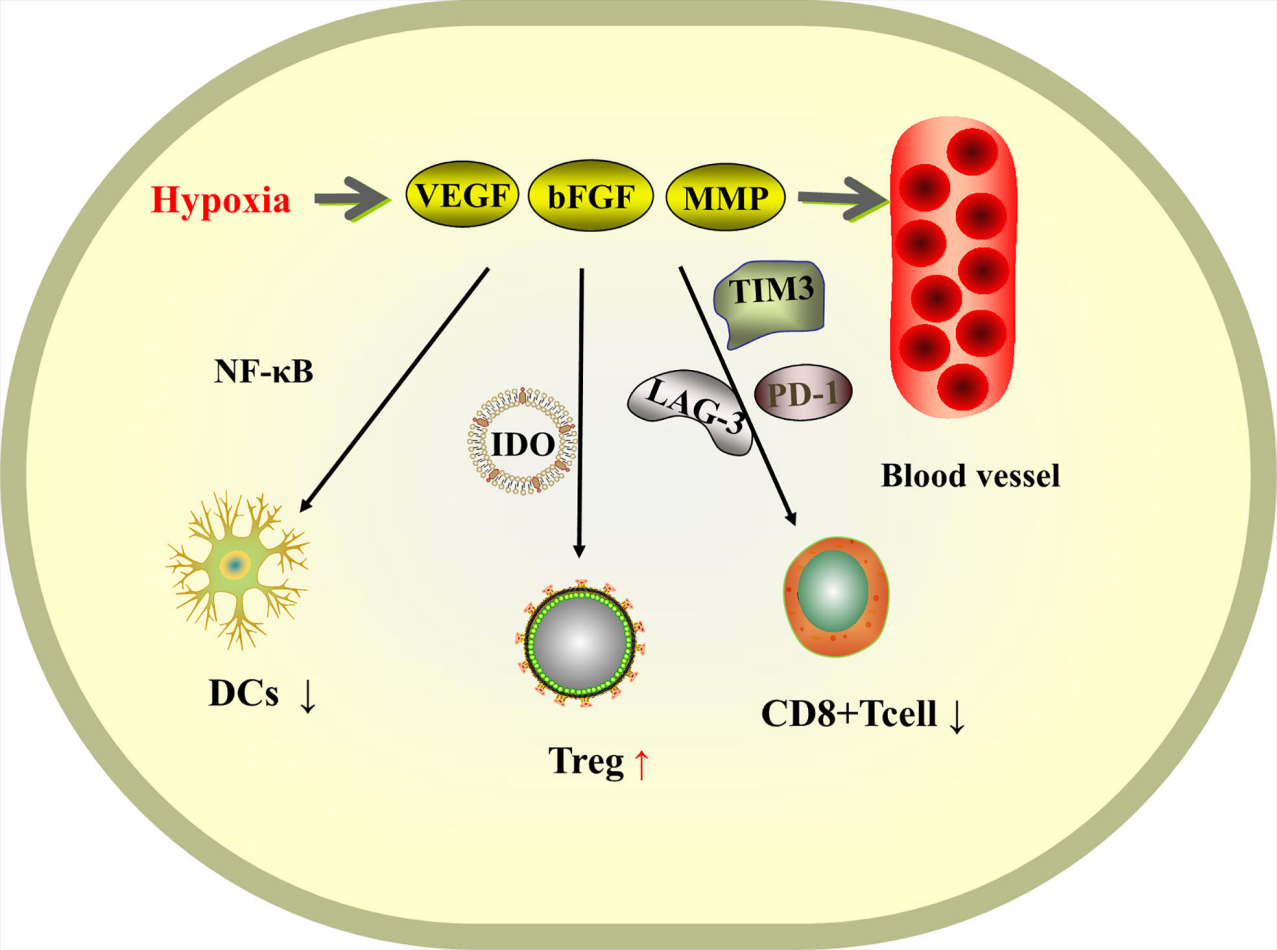
Schematic diagram of the mechanism of tissue hypoxia-induced VEGF-promoted tumor vascular proliferation and immunosuppression.

Hepatocellular carcinoma is a highly vascularized tumor. At the tumor site, hypoxia induces tumor cells and stromal cells to secrete a variety of pro-angiogenic factors, such as vascular endothelial growth factor (VEGF), basic fibroblast growth factor (bFGF), and matrix metalloproteinase (MMP) (82), leading to vascular proliferation, and the abnormally proliferating vessels provide tumor development providing nutrients for tumor development. The theory is that controlling the rate of angiogenesis so that tumor growth lacks nutritional support will slow down the growth of the tumor. The VEGF pathway is not only a key regulator of tumor angiogenesis, but also has the ability to inhibit the infiltration and function of cytotoxic T lymphocytes by affecting immune cells in the myeloid and lymphoid lineages (83). VEGF inhibits the maturation of dendritic cells (DCs) by activating NF-κB and suppresses the activation of T cells by promoting the production of indoleamine 2,3-dioxygenase (IDO), as well as the induction of Treg cells. VEGF also regulates immunity in hepatocellular carcinoma by inducing the expression of immunosuppressive receptors, including PD-1, lymphocyte activation gene 3, T-cell immunoglobulin and mucin domain 3 (82), promoting CD8+ T-cell failure and tumor escape free escape (**Figure 3**). Therefore, anti-angiogenic therapy can be an idea for the treatment of liver cancer. Anti-angiogenesis can induce normalization of tumor vascular structure, remove blood vessels necessary for tumor growth and metastasis, and also promote antigen presentation and activation of cytotoxic CD8+ t cells (84), reprogramming the tumor immune microenvironment (85) and transforming immunosuppression into immune stimulation, thus improving the immunosuppressive microenvironment of tumors.

However, anti-VEGF antibody monotherapy has failed to produce satisfactory antitumor efficacy in human HCC patients so far (84). Therefore, a combination of anti-angiogenic therapy and immunotherapy can be considered, where on the one hand immunotherapy enhances the efficacy of vascular endothelial factor inhibitors, on the other hand vascular endothelial factor inhibitors alleviate resistance to immunotherapy.

Atezolizumab (anti-PD-L1) and bevacizumab (vascular endothelial growth factor (VEGF) inhibitor) have been shown to be efficacious (86, 87), and their combination has demonstrated antitumor activity and safety in a phase 1b trial in patients with unresectable hepatocellular carcinoma. In patients with unresectable hepatocellular carcinoma, atezolizumab and bevacizumab had better overall survival and progression-free survival than sorafenib (28, 88), and the combination of atezolizumab + bevacizumab had longer progression-free survival than atezolizumab treatment alone (89).

Lenvatinib is a multitargeted inhibitor of multiple growth factor receptors, including vascular endothelial growth factor receptor (VEGFR), fibroblast growth factor receptor (FGFR), platelet-derived growth factor receptor (PDGFR), and the proto-oncogenes RET and KIT (90). Abnormally activated FGF signaling can directly drive cell proliferation and survival, promoting tumor angiogenesis and progression. Lenvatinib inhibits the vascular endothelial growth factor receptor and fibroblast growth factor bodies, and this dual-target inhibition effect enhances the antitumor activity of anti-Lenvatinib in HCC, while also strengthening the efficacy of PD -1 antibodies. A growing body of evidence suggests that Lenvatinib in combination with anti-PD-1 antibody significantly inhibits tumor growth *in vivo*, induces long-term immune memory, and has no significant adverse effects (91). Preliminary data from a clinical trial showed an objective remission rate (ORR) of 46% for Lenvatinib in combination with pembrolizumab (PD-1 antibody), with better response rates and duration of response (90). In July 2019, based on the results of KEYNOTE-524/Study 116 (NCT03006926), the FDA announced the approval of Lenvatinib in combination with pembrolizumab for the treatment of HCC (92). In addition, the efficacy of nivolumab and Lenvatinib has been confirmed, but more data are needed to validate (83).

It is worth noting that if anti-VEGF therapy causes excessive vascular pruning, it will aggravate tumor hypoxia, so we should reasonably apply anti-VEGF drug doses to normalize dysfunctional tumor vessels, improve tumor perfusion and alleviate tumor hypoxia (85).

## Discussion

As a serious global health problem with poor prognosis and high mortality rate, there has been tremendous progress in recent years in understanding the pathogenesis, early detection and diagnosis (93), staging and treatment of hepatocellular carcinoma (94). Research advances in the use of molecularly targeted agents (MTAs) and immune checkpoint inhibitors have significantly improved the prognosis of patients with this disease (95), demonstrating superior survival benefits, durable responses, and a manageable safety profile in advanced HCC. Oncolytic viruses, cancer vaccines (96), pericyte therapy (97), photothermal therapy (PTT) and photodynamic therapy (PDT) (98), and nanotechnology are also being explored. However, due to the specific immune tolerance of the liver (99) and the complexity of the tumor microenvironment, the treatment of hepatocellular carcinoma remains a great challenge, and continuous research, including single-cell sequencing, is needed in the future to explore new immunotherapeutic targets and personalized treatment protocols (100). In addition to this, the development of diagnostic, prognostic and biomarker prediction for hepatocellular carcinoma and other cancers using artificial intelligence is an exciting prospect (101). The role of menopausal hormones in reducing the risk of liver cancer still needs to be explored (102). With the development of science and technology and the advancement of research methods, the efficacy of treatment for liver cancer is also expected to be improved in the future.

## Author contributions

HW, FS, and LZ concepted and designed the review. FS, SZ and MZ wrote the manuscript. HW, ZP, LX, and LZ revised the manuscript. All authors contributed to the article and approved the submitted version.

## Conflict of interest

The authors declare that the research was conducted in the absence of any commercial or financial relationships that could be construed as a potential conflict of interest.

## Publisher’s note

All claims expressed in this article are solely those of the authors and do not necessarily represent those of their affiliated organizations, or those of the publisher, the editors and the reviewers. Any product that may be evaluated in this article, or claim that may be made by its manufacturer, is not guaranteed or endorsed by the publisher.
